# Design, Development, and Evaluation of an Injury Surveillance App for Cricket: Protocol and Qualitative Study

**DOI:** 10.2196/10978

**Published:** 2019-01-22

**Authors:** Najeebullah Soomro, Meraj Chhaya, Mariam Soomro, Naukhez Asif, Emily Saurman, David Lyle, Ross Sanders

**Affiliations:** 1 Broken Hill University Department of Rural Health University of Sydney Broken Hill Australia; 2 Rural Clinical School The University of Adelaide Adelaide Australia; 3 Discipline of Exercise and Sport Science, Faculty of Health Sciences The University of Sydney Lidcombe Australia; 4 Academy of Computer Science and Software Engineering University of Johannesburg Johannesburg South Africa; 5 School of Public Health and Community Medicine University of New South Wales Sydney Australia

**Keywords:** cricket, injury surveillance, mobile app, mobile phone, TeamDoc, mHealth

## Abstract

**Background:**

Injury surveillance and workload monitoring are important aspects of professional sports, including cricket. However, at the community level, there is a dearth of accessible and intelligent surveillance tools. Mobile apps are an accessible tool for monitoring cricket-related injuries at all levels.

**Objective:**

The objective of this paper is to share the novel methods associated with the development of the free TeamDoc app and provide evidence from an evaluation of the user experience and perception of the app regarding its functionality, utility, and design.

**Methods:**

TeamDoc mobile app for Android and Apple smartphones was developed using 3 languages: C++, Qt Modeling Language, and JavaScript. For the server-side connectivity, Hypertext Preprocessor (PHP) was used as it is a commonly used cross-platform language. PHP includes components that interact with popular database management systems, allowing for secure interaction with databases on a server level. The app was evaluated by administrating a modified user version of the Mobile App Rating Scale (uMARS; maximum score: 5).

**Results:**

TeamDoc is the first complementary, standalone mobile app that records cricket injuries through a smartphone. It can also record cricketing workloads, which is a known risk factor for injury. The app can be used without the need for supplementary computer devices for synchronization. The uMARS scores showed user satisfaction (overall mean score 3.6 [SD 0.5]), which demonstrates its acceptability by cricketers.

**Conclusions:**

Electronic injury surveillance systems have been shown to improve data collection during competitive sports. Therefore, TeamDoc may assist in improving injury reporting and may also act as a monitoring system for coaching staff to adjust individual training workloads. The methods described in this paper provide a template for researchers to develop similar apps for other sports.

## Introduction

Emerging technologies are enabling new opportunities for science and medicine within high-performance sports [1]. High-performance sports benefit from applications of science, in the form of physiology, psychology and use of technology to monitor parameters of performance and injury [2,3]. In terms of technology, an emerging domain is mobile health (mHealth), which involves mobile computing by the use of apps on smartphones to improve health [4]. mHealth to monitor an athlete’s health has also been identified as an area that can revolutionize sports medicine [5]. Electronic injury surveillance and monitoring tools (including mobile apps) are being used to monitor and predict injuries for sports including athletics, football, and handball [6-8]. However, their use in cricket is limited to elite players, with limited or no availability at the community level.

Cricket Australia (CA) uses the Athlete Management System for workload monitoring and injury reporting of their contracted players [9,10]. This system allows coaches and medical staff to monitor individual workloads, which may reduce the occurrence of overuse injuries. CA’s 10-year injury report (2005-2014) indicated that the 2013-2014 season had the lowest prevalence of injury (10.8%) compared with the 10-year average of 11.9% [10]. One factor contributing to the lower injury prevalence during the 2013-2014 season was the introduction of mandatory use of the Athlete Management System [10]. Mandatory reporting by players on variables such as sleep and workload may have helped them adhere to the recommendations of the team’s medical staff and, thereby, minimize reportable injuries.

However, the statistics for cricket injuries at the junior level tell a different story; a 5-year investigation of injuries in elite junior cricketers in South Africa indicated that 27% of the cricketers sustained injuries when only time-loss injuries were considered [11]. In Australia, however, the injury incidence in under-14 and under-16 players at the club level was 14.2%, despite the inclusion of both time-loss and nontime loss injuries [12]. Other studies have reported the injury incidence to range between 24% and 34%, and cricket-related musculoskeletal pain has been reported by 80% of school-aged cricketers in a season [13-17]. Yet, the actual injury burden may be higher than what is currently reported because most cricket injury reports discount the burden of nontime loss injuries, where the players continues to play despite the injury. Recording nontime loss injuries is now considered essential according to injury epidemiologists in other sports [7]. Therefore, in 2016, the new consensus statement for injury surveillance in cricket included the reporting of nontime loss injuries [18].

Adolescent athletes are susceptible to injuries due to rapid bone growth and musculoskeletal immaturity [19]. Evidence shows that increasing workload increases injury risk [15,20]. To tackle this problem, international cricket associations have proposed workload guidelines [21,22]. However, these are not being extensively followed at the junior level and may be attributed to the lack of support staff to keep track of the bowling and batting workloads and injuries [23].

Monitoring the training workload by using subjective measures from an athlete is an effective way to address the issue of training loads in sports such as cycling, athletics, and football [24-27]. Similarly, if cricketers record their workloads with a user-friendly mobile app, the increased surveillance may allow coaches to devise injury prevention strategies. Currently, no free-to-download mHealth apps are available that can record cricket-related injuries and monitor workload. Given that elite cricketers emerge from junior cricket, it would be logical to implement such a system at the junior or amateur level. This would have several benefits: first, it could reduce the possibility of talented cricketers being “lost” from the player pool because of injury. Second, it could provide exposure and experience with reporting injuries and workload for those cricketers who progress to the elite levels, where reporting is mandatory. Finally, reporting injuries may enable players to seek timely medical advice and minimize injury effect.

The primary aim of this paper is to outline the methods for app development used to design TeamDoc, a free mHealth app providing paperless, user-friendly solution for monitoring injuries and workloads in junior cricket. The sharing of novel methods associated with the development of TeamDoc will act as a foundation for future app developments in the area of injury surveillance and workload monitoring. The secondary aim of the paper is to provide evidence from a pilot evaluation of the user experience, functionality, utility, and design of the app. As end-user perceptions have been shown to be an important aspect for the long-term uptake of new interventions [28], behavior change was also appraised. The results of the evaluation will assist in improvement of future apps in this domain.

## Methods

### Software System Development

When designing the app, we took into account several important considerations. First, the app should ensure confidentiality of the data provided by the players. Second, the system needs to be user friendly with ease for quick data entry (not exceeding >2 minutes). Third, there needs to be a back-end server that stores the data for future analysis. Fourth, it should be usable and adaptable for common operating systems. Finally, the injury and workload data must be presented in a way that is easily read and interpreted.

**Figure 1:**
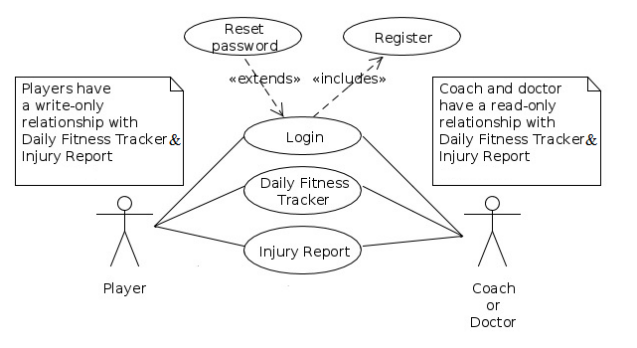
A Unified Modeling Language use-case diagram details the functionality offered to players and coaches.

We divided the TeamDoc software system design into 3 components: player interface, coach interface, and a back-end system to securely store the data. The player and coach interfaces are completely separate. This design protects player privacy, as the player interface only permits authorized players to log the data. Similarly, the coach interface only allows authorized coaching staff to access the data. The design of the software relies on client-server architecture, with the player and coach interfaces operating as clients (resource and service requesters) and the back-end system operating as the server (resource and service provider; Figure 1).

### Tools and Languages

#### Client-Side Software

The client-side software used was an open-source software development platform, Qt 5.3 (Qt Company Ltd, Finland, 2014). This platform was chosen because of its cross-platform compatibility (ability to work on multiple operating systems; eg, it supports Windows, Mac OS X, and Linux for desktops and Android, iOS, and Windows Phone for mobile phones) [29]. This meant that even though the initial release was compatible only with Android, the source code can be ported to 15 other operating systems with relative ease [30]. In addition, it allows developers to program software using a range of different programming languages. We used 3 languages—C++, Qt Modeling Language (QML), and JavaScript—because they are well documented and supported by Qt 5.3.

The chosen platform and languages simplify the construction of custom user interfaces (UIs) and provide the opportunity to augment UI components with high-level logic. The Qt software development kit was used to develop the client-side software. This integrated development environment had a compiler for the C++ language and a graphical user interface designer, allowing for rapid prototyping of UIs.

#### Server-Side Software

The most important considerations for designing the server-side software were data security and cross-platform connectivity. Hypertext Preprocessor (PHP) scripting language was used as it allows cross-platform server-side connectivity, and it is also commonly used in the website development. PHP includes components that interact with popular database management systems and provides protection against certain malicious attacks, such as structured query language (SQL) injections, which is a technique used to exploit data from servers (although this must be explicitly instructed in source code), allowing for secure interaction with databases on a server level.

The data entry required to compile injury reports was quite simple, and this led to TeamDoc being built with a thin-server architecture, which is a type of architecture suitable for systems where the majority of computation occurs on the client side, as opposed to fat-server architecture, which requires higher computational resources on the server side. PHP was chosen instead of native executable code, as it is more suitable given this thin-server architecture. Alternatively, with native executable code, Transmission Control Protocol server sockets would have to be implemented and socket communication handled, which would have increased the complexity of this system.

#### Software Architecture

**Figure 2:**
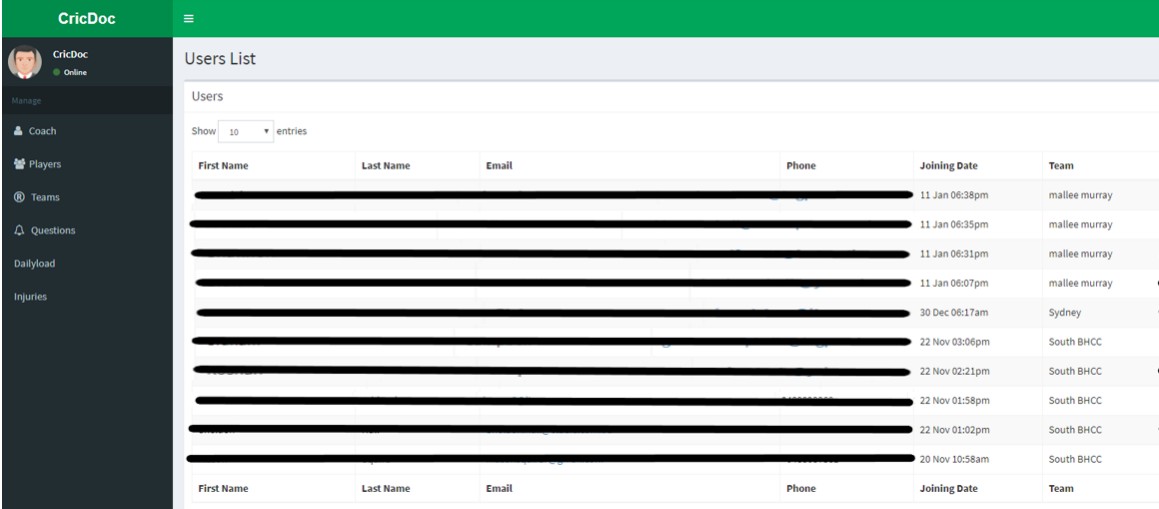
Screenshot from server interface showing data view available to the coaching staff.

The 3 components of the system were as follows:

1. *Player App*: A write-only function permits only data entry and restricts access to individual output data, thereby ensuring data privacy. Players can submit information that will be compiled into the Daily Fitness Tracker and Injury Report System.
2. *Coach App*: A read-only function limits data access so that no amendment can be made by the coaching staff after the data have been recorded by the player. This ensures data security and authenticity as multiple people may be involved in a team’s coaching staff. Only coaching staff (including doctors) can visualize the collected information, both graphically within the app and in the tabular form (via a Comma-Separated Values file compatible with Microsoft Excel and other spreadsheet apps; Figure 2).
3. *Server-side operations*: Both players and coaches or doctors can log-in and register, and if necessary, reset their password.

The interaction of a design-focused language, QML, and 2 logic-focused programming languages, C++ and JavaScript, allowed for the use of the Model-View-Controller (MVC) software architectural pattern. MVC supports the UI, internal data representation, and logic of the project to be independent so that a change in one component does not directly affect the next one [31]. This architectural pattern also ensured that team members involved in the project design were able to engage with the project from their preferred aspect—design, logic, or database management—and minimized delay with parallel, rather than sequential development and implementation.

#### Database Implementation

To improve cross-platform connectivity in the future, we used a popular and robust open-source database management system called MySQL to capture, query, and administer the data collected by the TeamDoc app. The database comprises 5 tables that store raw and processed data:

- The *Users table* contains information about every user, including user-identification and password.
- The *ResetPasswords* table is an administrative table that stores computer-generated temporary passwords for users who have forgotten their passwords.
- The *DailyFitnessTracker* table stores processed final scores as well as the raw values input by players.
- The *InjuryReport* table collects the players’ responses and can be accessed by coaches and affiliated medial staff.
- The *Attendance* table stores player attendance and injury details for a 40-week season, each week includes 3 practice sessions and 1 match. These variables may be adjusted as needed.

The information for each table is linked to each player through a user ID and time-stamp of submission. The values in the tables can be queried manually through SQL or through the interfaces available to coaches and doctors.

### User Interface Design

**Figure 3:**
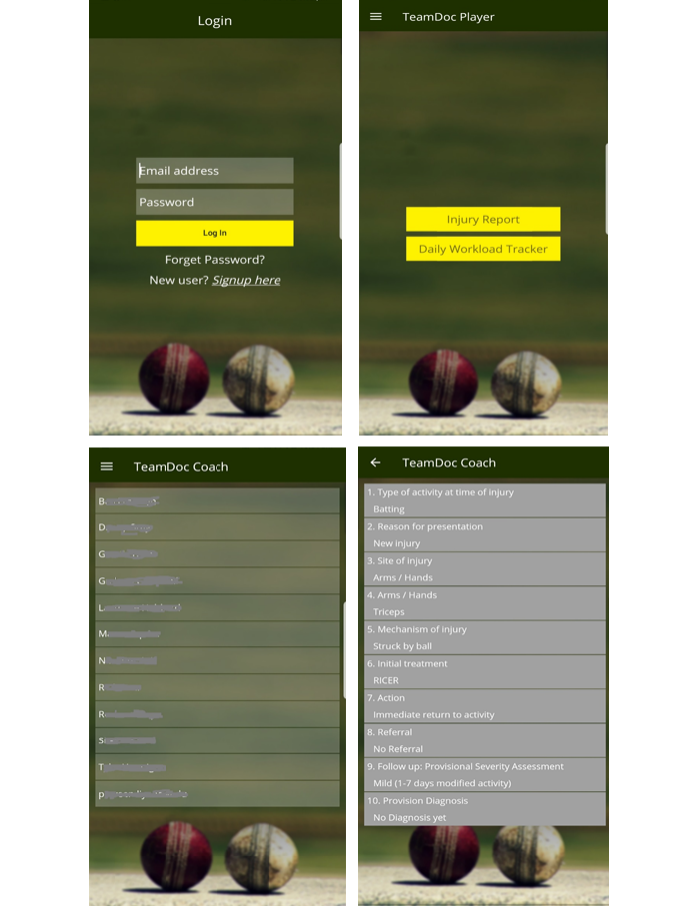
User interface of the TeamDoc app. Top left: log-in view; top right: main tab of the player app; bottom left: main tab of the coach app; bottom right: injury report tab in the coach app.

The UI of TeamDoc was designed to be user friendly with little-to-no training time. Figure 3 illustrates the log-in and player and coach interfaces. The player interface provides forms with text-input-boxes, numeric sliders, check-boxes, and radio buttons to make data input quick and simple (Multimedia Appendix 1).

#### Injury Reporting

**Figure 4:**
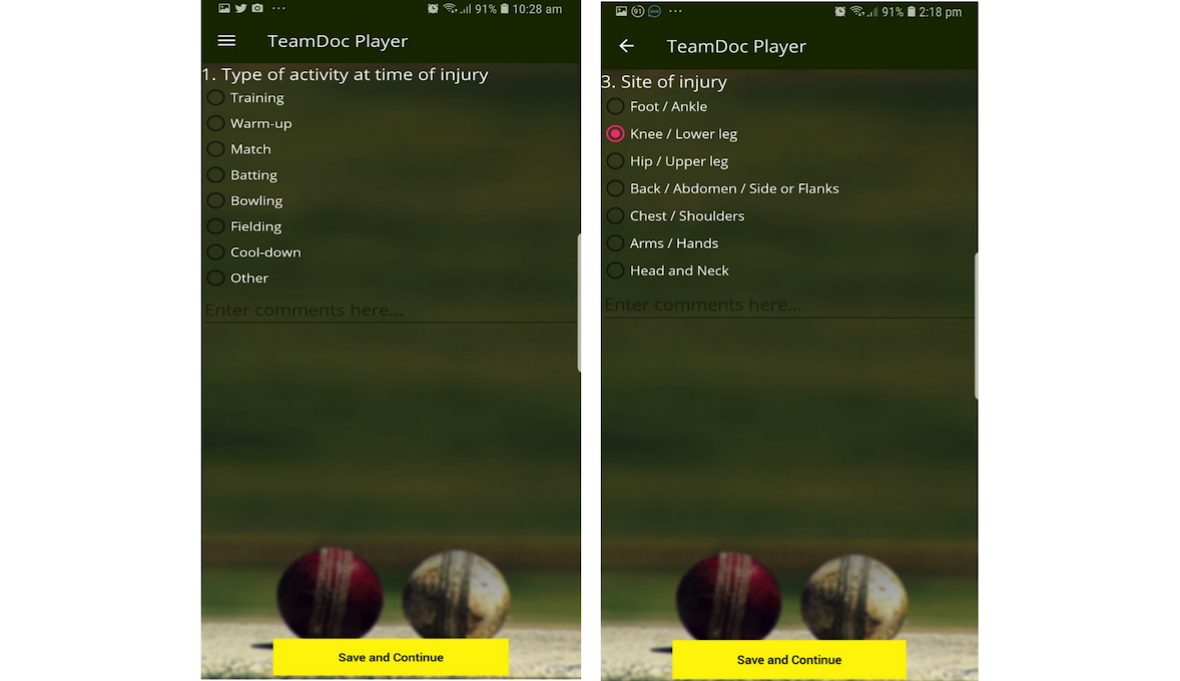
User interfaces for the injury reporting tab in the TeamDoc player app.

The injury reporting in the UI was based on the standard injury reporting form developed by Finch et al and used by the Sports Medicine Australia [32,33]. The form had questions on the activity at the time of injury, reason for presentation, site of injury, nature and mechanism of injury, initial treatment given, action taken after the injury, referral, etc. The site of injury function uses a “branching” logic, which means that when a specific body region was indicated as injured, only questions related to that region would appear. For example, if *Knee* or *Lower leg* was selected, then options such as calf muscles, knee joint, etc, came up. Figure 4 shows injury reporting forms in the player’s interface; Multimedia Appendix 1 shows all UIs in the players’ app, and Multimedia Appendix 2 shows a video run-down of the app.

#### Workload Reporting

Workload monitoring was designed for batting and bowling. For batting, the number of balls batted was the primary input and the number of balls bowled for bowling. CA’s fast bowler workload guidelines were used to determine if a fast bowler overbowls or underbowls [21]. These guidelines are part of the coach training programs and are standard to monitor the training of fast bowlers. All input from the players is stored on the server and is accessible for the doctor and coach at any time.

### Mode of Availability of Software

We built the client side to simplify porting it to diverse smartphone operating systems, the initial release for the Android platform. In addition, we implemented the server side of TeamDoc to be compatible with various server operating systems and avoid dependence on a single technology. This consideration made the release to Apple’s iOS platform.

### App Evaluation

We used a modified user version of the Mobile App Rating Scale (uMARS) to critically appraise the app [34]. uMARS is derived from MARS, which is a validated mobile app evaluation tool and has been used extensively to rate the quality of medical-based apps [35-37]. It comprises 31 questions, mostly using a Likert-type rating scale, to evaluate an app on the following 3 domains: (1) Quality score: it examines engagement, functionality, aesthetics, and content information provided in the app; (2) Subjective quality: it questions likelihood of recommending the app to others, use in future, overall rating, etc; and (3) Behavior change: it assesses the perceived impacts on knowledge, attitude, awareness, and behavior. The internal consistency (alpha=.90) and interrater reliability (.79) for MARS is acceptable [35].

The MARS was modified by excluding 7 questions on the information content of the app. These questions are only relevant for apps that provide content to users. The 24 questions relevant to assess the TeamDoc app were used (see Multimedia Appendix 3). Field testing and validation of the app was conducted during the initial phase of development by collecting informal user reviews from 20 players on the University of Sydney Cricket team. After the final launch of TeamDoc on the Android and iOS app stores, 8 registered cricket clubs in New South Wales and Victoria, Australia, were invited to use the app during the 2017-2018 season using convenience sampling.

### Procedure for User Version of the Mobile App Rating Scale Administration

We administered the modified uMARS at the end of the season through a Web-based survey using the Research Electronic Data Capture (REDCap) Survey instrument. REDCap is a secure, Web-based app for building, disseminating, and managing Web-based surveys and complies with the Health Insurance Portability and Accountability Act regulations. Participant information was secure and only available to the authors through the use of both server authentication and data encryption using secure Web authentication, data logging, and Secure Sockets Layer. The survey was available on Web, and an invitation with the survey link was sent to the registered users.

### Data Analysis

We exported the survey results to Microsoft Excel v2013, and performed basic calculations to report standard descriptive statistics. For qualitative data, we performed the content analysis by categorizing the content into themes. Top 3 themes from each category were reported.

## Results

### Participants

In this study, 3 of the 8 club teams agreed to participate in the app testing and evaluation. Each club team had an average squad size of 14 players. In total, 42 club cricketers (14×3) registered and used the app. The data collected by the app were verified with the data stored on the server by the developers, and the results indicated 100% data accuracy.

App review using the modified uMARS was completed by 16 of 42 cricketers (38% of the app user base). All the respondents were current club cricketers with a mean age of 27.4 (range 16-42) years. Of all, 9 users were running the app on Android smartphones and 7 on Apple iOS (6 used an iPhone and 1 used an iPad). No coaches or doctors responded to the survey.

### User Version of the Mobile App Rating Scale Ratings

**Figure 5:**
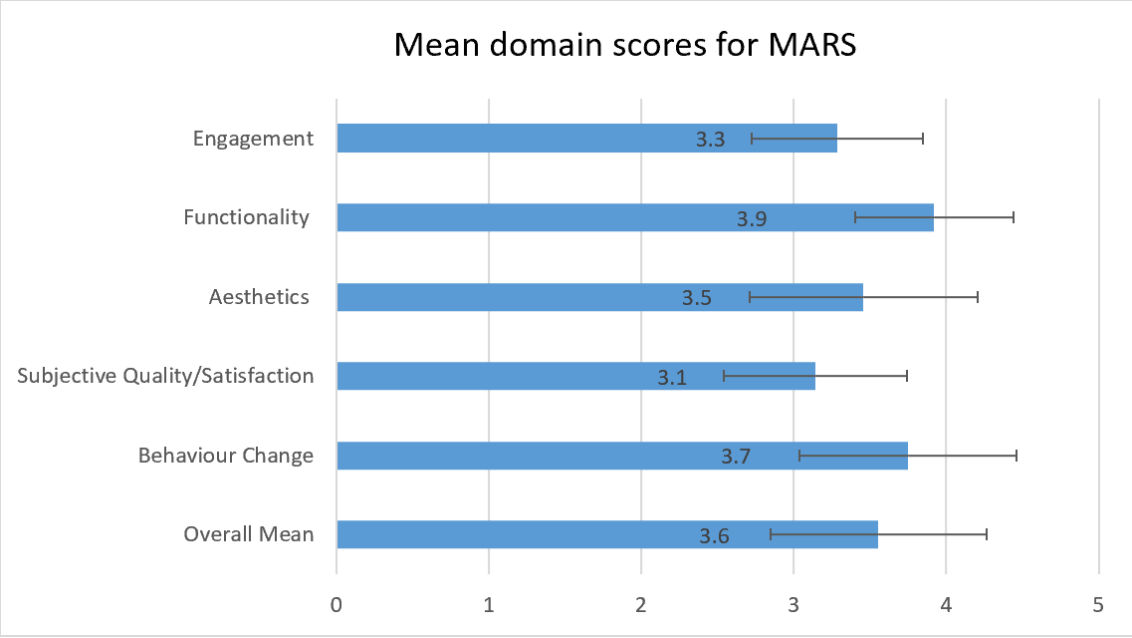
Mean (SD) domain scores for the Mobile App Rating Scale (MARS).

The mean app quality score (maximum score=5) was 3.6 (SD 0.6); this was compiled from the mean scores on app functionality, engagement, and aesthetics. The mean subjective quality score was 3.1 (SD 0.7). Behavioral change, which included an assessment of the perceived impacts on knowledge, attitude, awareness, and behavior, had a mean score of 3.8 (SD 0.5). The overall mean uMARS score (maximum score=5) was 3.6 (SD 0.5), and the scores ranged from 2.9 to 4.8 (Table 1 and Figure 5).

- *Engagement.* This score ranged from 2 to 4.4 (mean 3.3 [SD 0.7]). Engagement scores were compiled from 5 questions on entertainment, interest, customization, interactivity, and appropriateness for target audience. Appropriateness for target group was rated highly among the “engagement” questions, with an average score of 3.8 [SD 0.9]. However, customization received the lowest score (mean 2.94 [SD 1.1]).
- *Functionality* score ranged from 2.3 to 5 (mean 3.9 [SD 0.7]). Functionality scores were compiled from 4 questions on performance, ease of use, navigation, and gestural interactivity. Ease of use scored highly within the category (mean 4.1 [SD 0.6]), while gestural interactivity was the lowest-rated category with a mean score of 3.8 (SD 1.0).
- *Aesthetics* scores ranged from 2.3 to 4.3 (mean 3.5 [SD 0.6]) and were for questions on the app’s layout and graphics to the visual appeal. The layout of the app had the highest score (mean 4.2 [SD 1.3]), and visual appeal had the lowest score (mean 3.2 [SD 0.8]).
- *Subjective Quality* or *Satisfaction.* This score ranged from 2.25 to 4.75 (mean 3.14 [SD 0.7]). These scores were for questions on a recommendation to others, use in the next 12 months, overall star rating, and paying for the app. Recommendation to others received the highest score (mean 3.75 [SD 1.0]), while paying for the app received the lowest score with a mean of 2.75 (SD 1.2).
- *Behavior Change* scores ranged from 2.7 to 5 (mean 3.6 [SD 0.5]). These scores were from 6 questions about awareness, knowledge, attitudes, intention, behavior to change, and help-seeking. The question on behavior change describing the likelihood of the app in improving the understanding of injury and seeking help for it received the highest score (mean 3.9 [SD 0.7]). Conversely, the question on the role of the app to improve the knowledge about injuries received the lowest score (mean 3.6 [SD 0.9]).

### User Perceptions

User perceptions were collected with 2 open-ended questions: (1) If you decide or decided not to use this app, what will be the possible reasons for it? (2) What improvements do you want to see in the future versions of the app?

Majority of the respondents (n=10) were not currently using the app (nonusers). The main reasons for not using the app were UI, time consumption, and forgetfulness. Users expressed the importance of functional design improvement of the app, which may have made them feel they were spending too much time on filling out information. Several users expressed that the app lacked the graphic interface and breadth of content to engage them for regular use.

I like using this app, however, I need more interactive options in it such as scores and health tips etc.

Lack of feedback, unable to enter data in days after activity

Time consuming

Most current users (n=6) mentioned that the reasons for future disuse will be if they did not get injured, stopped playing cricket, or forgot using it.

Due to no injuries

If I don’t play in the future

The only reason I wouldn't (use the app) would be forgetting to.

On the question regarding future improvements in the app, the main reasons cited by current nonusers (n=10) were linked to lack of feedback, UI, and user experience.

Being able to see how the data is collated and be able to refer back to this data would help with the information entered. Entering data two days after activities by putting in date (not rely on entering data immediately after activity) would allow more entries to be input. Workload app would consider multiple activity types for example, running while not playing cricket, gym time etc.

No graphics or engaging content

Reviews of current app users (n=6) identified two main themes where improvements in the future versions could be made, that is, improvement in the interactivity and content and improvement of UI.

Injury reports should be graphically displayed rather than plain text.

User-design can be improved my making the content more interactive.

I would like to see more information diet, such as calorie tracker, dietary recommendations before and after the game etc.

**Table 1:** Mobile App Rating Scale ratings for the TeamDoc app.

Subject	Engagement	Functionality	Aesthetics	Subjective quality or satisfaction	Behavior change	Overall mean
1	3.6	4	3.3	4	3.7	3.8
2	4.2	5	4.3	2.8	4.2	3.8
3	4.4	4	4.3	3.8	4	4.0
4	2.6	4	3.3	2.8	2.7	2.9
5	3	2.3	2.3	2.8	3.5	2.9
6	2.8	3.5	3.7	2.3	3	2.9
7	2.6	2.8	2.7	4	4	3.6
8	4.4	4.8	4.3	4.8	5	4.8
9	3.4	4.8	3.7	3.3	4	3.8
10	3.8	3.5	3.3	3	4	3.5
11	3.6	4	4	3.3	3.8	3.7
12	2.6	4	3.7	2	3.7	3.0
13	2.8	4.5	2.7	3.8	3.8	3.6
14	2	4	3.3	2.3	3.3	2.9
15	3.6	4.3	3	2.8	3.5	3.3
16	3.2	3.5	3.3	3	3.8	3.4
Mean (SD)	3.3 (0.7)	3.9 (0.7)	3.5 (0.6)	3.2 (0.7)	3.8 (0.5)	3.6 (0.5)

### Validation of Design Considerations

First, data confidentiality was assured because none of the test users were able to access the other player’s records without validated authentication details. Second, the system’s user-friendliness was validated with high mean “functionality” and “ease of use” scores of 3.9 and 4.1, respectively. However, on average, users took 3 minutes to fill out the injury and workload entries, so the time efficiency for data entry did not meet the aim of 2 minutes per entry. Future versions should reduce the number of data entry fields and make the entry more engaging to enhance user compliance and entry efficiency. The third consideration was data storage for future analysis. The testing and validation showed that data output through the server was 100% accurate and could be retrieved instantaneously; this was tested by the investigators by asking the players to enter data on the player app and then cross-verifying the data with the player after downloading the information from the server. The fourth consideration was app’s cross-platform availability, and the app was made available for both Android and iOS operating systems. Finally, the UI for injury and workload data entry was to be presented in a way that was easy to input and interpret. This was validated during user-rating functionality and had an overall mean uMARS score of 3.8. Most features of the app scored >3 out of 5 on uMARS domains, showing an overall end-user satisfaction.

## Discussion

### Principal Findings

It is a common perception that cricket is a nonimpact sport associated with fewer injuries than other sports. However, the literature shows that when injuries are measured in terms of injury rates (ie, per hour of athletic exposure), junior and amateur cricketers have higher rates of injury than professional cricketers [38], and injury rates are comparable to other noncontact or quasi-contact team sports such as soccer, basketball, and tennis [39]. In recent times, the use of mHealth and Web-based technology to monitor an athlete’s health is becoming an important component of sports medicine [5]. The development of the TeamDoc app was inspired by this concept and is the first standalone mobile app that can record injuries in cricket through a smartphone without the need for connectivity from parent software on computers. The injury questionnaire was tailored to effectively cater cricket-related injuries, for example, a finger injury while catching the ball.

The benefits of using electronic injury surveillance systems have been extensively documented by Karlsson [6]. It identifies the advantages of using such systems compared with a paper-based system as having less risk of error while transcribing and minimal to no logistic issues. TeamDoc provided the functionality to store the injury data on the server, thereby delivering a paperless solution for tracking injuries and providing ease of access for the team’s coaching staff to track injury profiles of the players. Yet, of 8 club teams, 5 (63%) declined to participate when invited to use and evaluate the app. The two main reasons cited by the team coaches or captains for not participating were “time commitment” and “injury not a major issue for the team.” To change these perceptions, educating the players and coaches about cricket-related injuries, injury prevention strategies, and the role of technology to prevent future injuries is important.

The overall mean uMARS score was 3.6 out of 5. This is comparable to the mean score for other health-related surveillance apps; for instance, a review of 7 apps for prostate cancer risk calculation had a mean quality score of 3.75 [36]. Similarly, a review of 20 epilepsy self-management apps found a mean quality score of 3.25 [37]. The results indicated that of the 16, only 6 (38%) respondents were current users. The high rate of attrition may be linked to the low scores on satisfaction on the subjective quality of the app (3.14/5) and engagement (3.3/5). The main reasons for the low score on engagement were that half of the users rated the app having no or very basic interactive features and a quarter rated the app as boring. Previous research has shown that providing feedback to users and considering their preferences are important aspects when introducing new injury prevention strategies [28,40]. Therefore, an understanding of perceptions and behaviors when adopting new technology for injury prevention is important. Escoffery et al advocated that for apps targeting behavioral change, developers should work with behavioral scientists to improve the engagement features within the app and encouraged the use of theoretical strategies for behavior change during conceptualization and design phases of app development [37].

The mandatory reporting of injuries and workload by the players may be another reason for high attrition rate from regular use of the app. Medical professionals often use the terms “compliance” and “adherence” to describe the rate at which patients follow their “requests, commands, orders, or rules” [41,42]. These rules and orders can range from following the advice on talking medications and performing investigations to engaging in physical activity, etc. When patients fail to perform the required tasks, they are deemed to have poor compliance or adherence. More recently, “concordance” rather than compliance or adherence has been proposed to be a better alternative when dealing with certain populations [41]. Concordance in medicine is defined as “a state of agreement” between the patient and the physician [43]. Similarly, in sports, concordance can be inferred as a state of agreement between the player and the coach. In medicine, low concordance has been shown to have poor outcomes in patient satisfaction and perception of care [43]. Therefore, before introducing mandatory reporting or asking players to report their injuries and workload, educating them on the benefits of reporting may improve concordance and improve the uptake of the app in the future.

The reason for the low mean subjective quality score (3.18/5) can be attributed to a low score (2.8/5) on “would you pay for this app?” Only 2 users indicated that they would be willing to pay for the app in the future. The app was not designed for commercial use, but user inclination to pay for it may be a surrogate for their perception about the value of the app. “Lack of feedback” to users may be another issue for the low ratings. This is associated with the design constraints of the app, which only allows the coaches to view the data entered by players. Previous research has shown that providing feedback to users is important to maintain their adherence while using Web-based injury surveillance systems [44]. In future versions, it may be useful to allow players a view of their own data so that they can track their activity levels and set up goals. Another feature that may be useful to improve user experience is the inclusion of “gamification” and “social media plugins.” Gamification features may include features such as players setting up weekly targets for their activity and getting rewards if they achieve their target. Social media and sports news plugins may improve user experience and encourage regular use of the app.

There were multiple limitations within the current version of the app. For example, there was no mechanism for alerting fast bowlers or coaches if a player exceeded age-related bowling workload recommendations nor mechanisms for delivering reminder alerts if players forgot to key in their workloads. However, important design considerations, such as security and confidentiality of data, were ensured by designing the app on PHP, which provided protection against malicious attacks by hackers, and by designing separate app interfaces for players and coaches. Another consideration during the development was cross-platform connectivity with other eHealth platforms on the client side of the app to simplify porting it to diverse operating systems.

### Conclusions

The use of mHealth in sports medicine can assist in wireless data capture that may be used to make informed, evidence-based decisions. TeamDoc follows this concept by allowing the coaching staff and the players to record data on injury and workload on the go. The app may assist coaches to make informed decisions in real time during match conditions. TeamDoc is available for free, which means that community-based clubs can access and use it. This study provides a guide to the architecture and framework for developing an injury surveillance and workload monitoring mobile app, which can be applied to design similar systems for other sports. The results from the user survey indicate that future versions of the app should have improved UI and interactivity features.

### Practical Implications

The following are the practical implications of the study:

- The ease to use the app “on the go” may mean better reporting of injuries at the junior level.
- The app can act as a monitoring tool for the coaching staff to adjust individual training loads for players, which may assist in reducing injuries.
- The methods of development used for this app can be applied by researchers and developers to introduce similar apps for other adolescent team sports.
- In the future, surveillance apps should focus on improved UI and interactivity to attract and retain users.

## Appendix

Multimedia Appendix 1 User Interfaces for the TeamDoc player App.

Multimedia Appendix 2 A video showing features of the player app.

Multimedia Appendix 3 User version of the Mobile App Rating Scale (uMARS) survey tool used for evaluation.

## Abbreviations

CA: Cricket Australia
uMARS: user version of the Mobile App Rating Scale
mHealth: mobile health
MVC: Model-View-Controller
PHP: Hypertext Preprocessor
QML: Qt Modeling Language
SQL: structured query language
UI: user interface
