# Supplementary material for: Design, Development, and Evaluation of an Injury Surveillance App for Cricket: Protocol and Qualitative Study
Source: JMIR Mhealth Uhealth. 2019 Jan 22;7(1):e10978. doi: 10.2196/10978 (PMC6362388; doi:10.2196/10978)
Supplement: Multimedia Appendix 1 [file mhealth_v7i1e10978_app1.pdf]

**Appendix 1 – User Interfaces for the Team Doc Player App.**

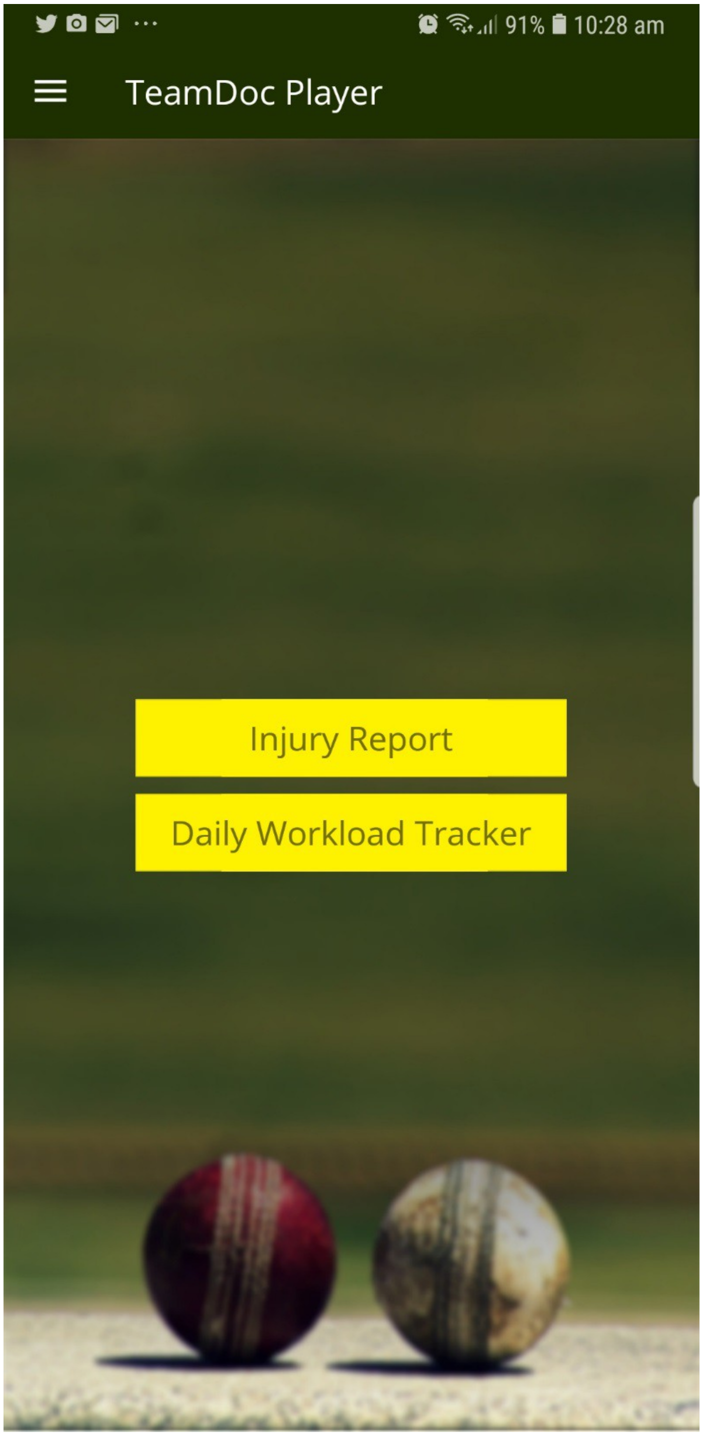

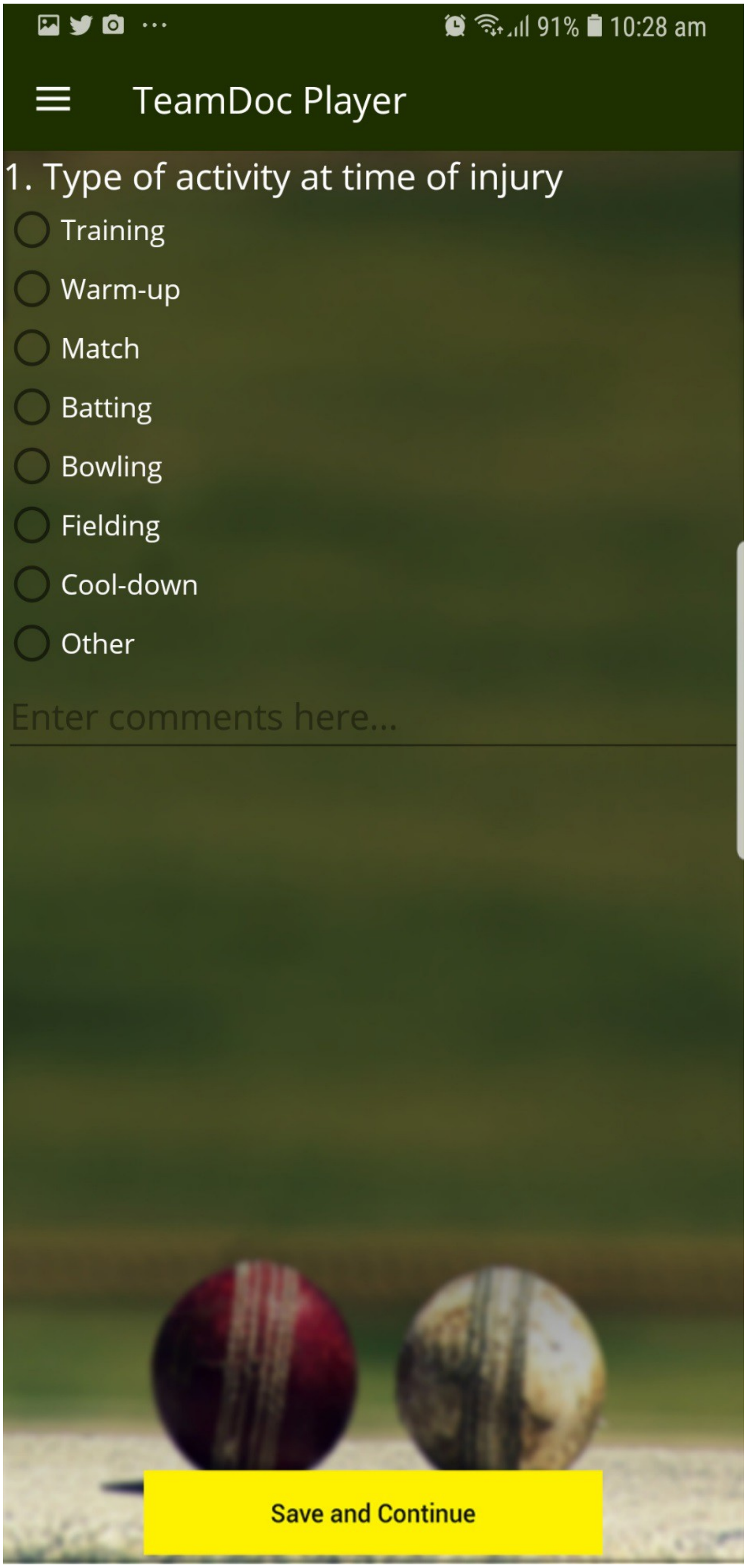
91% 10:28 am
TeamDoc Player
1. Type of activity at time of injury
Training
Warm-up
Match
Batting
Bowling
Fielding
Cool-down
Other
Enter comments here...
Save and Continue

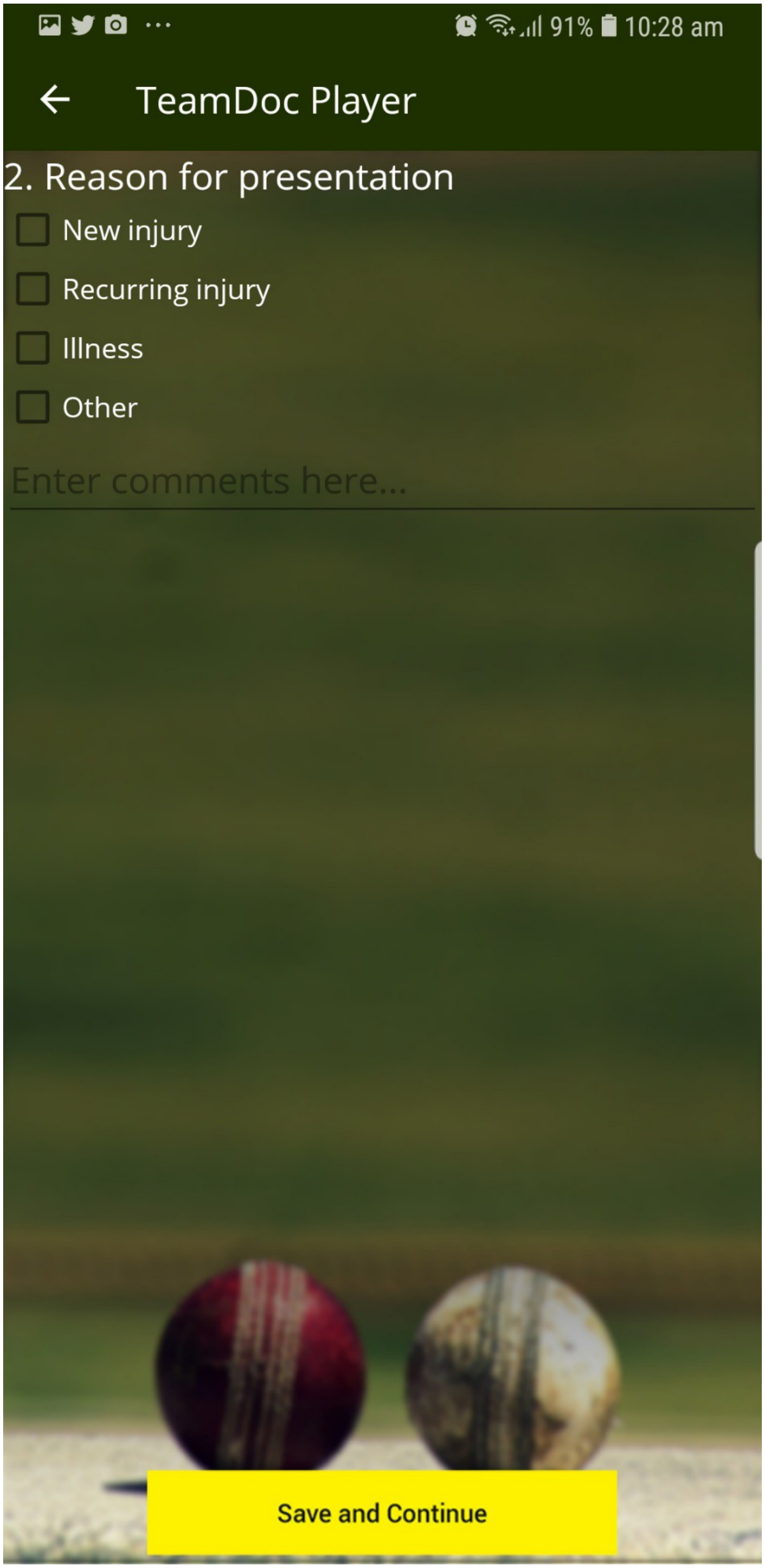
91% 10:28 am
TeamDoc Player
2. Reason for presentation
New injury
Recurring injury
Illness
Other
Enter comments here...
Save and Continue

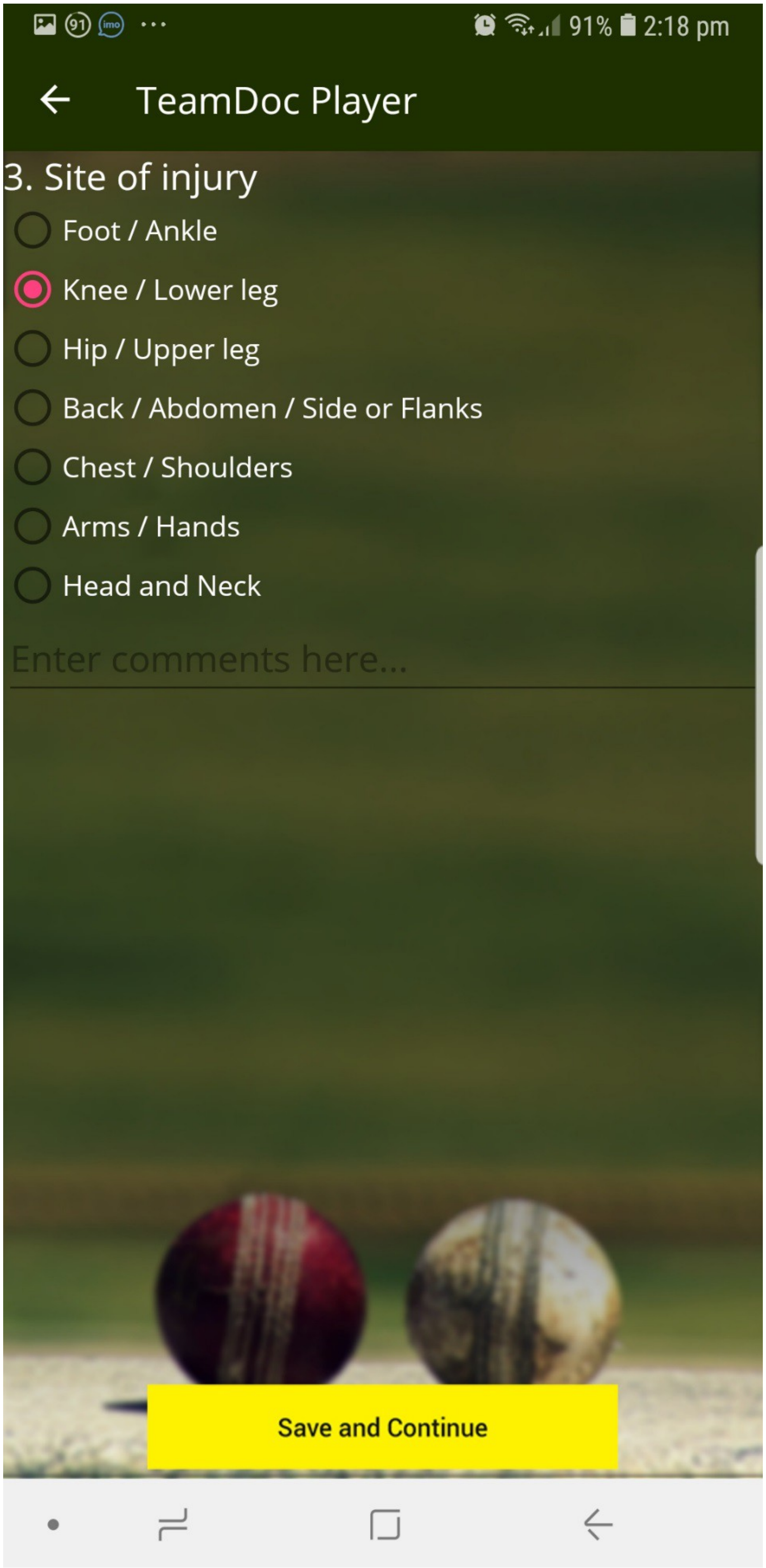
91% 2:18 pm
TeamDoc Player
3. Site of injury
Foot / Ankle
Knee / Lower leg
Hip / Upper leg
Back / Abdomen / Side or Flanks
Chest / Shoulders
Arms / Hands
Head and Neck
Enter comments here...
Save and Continue

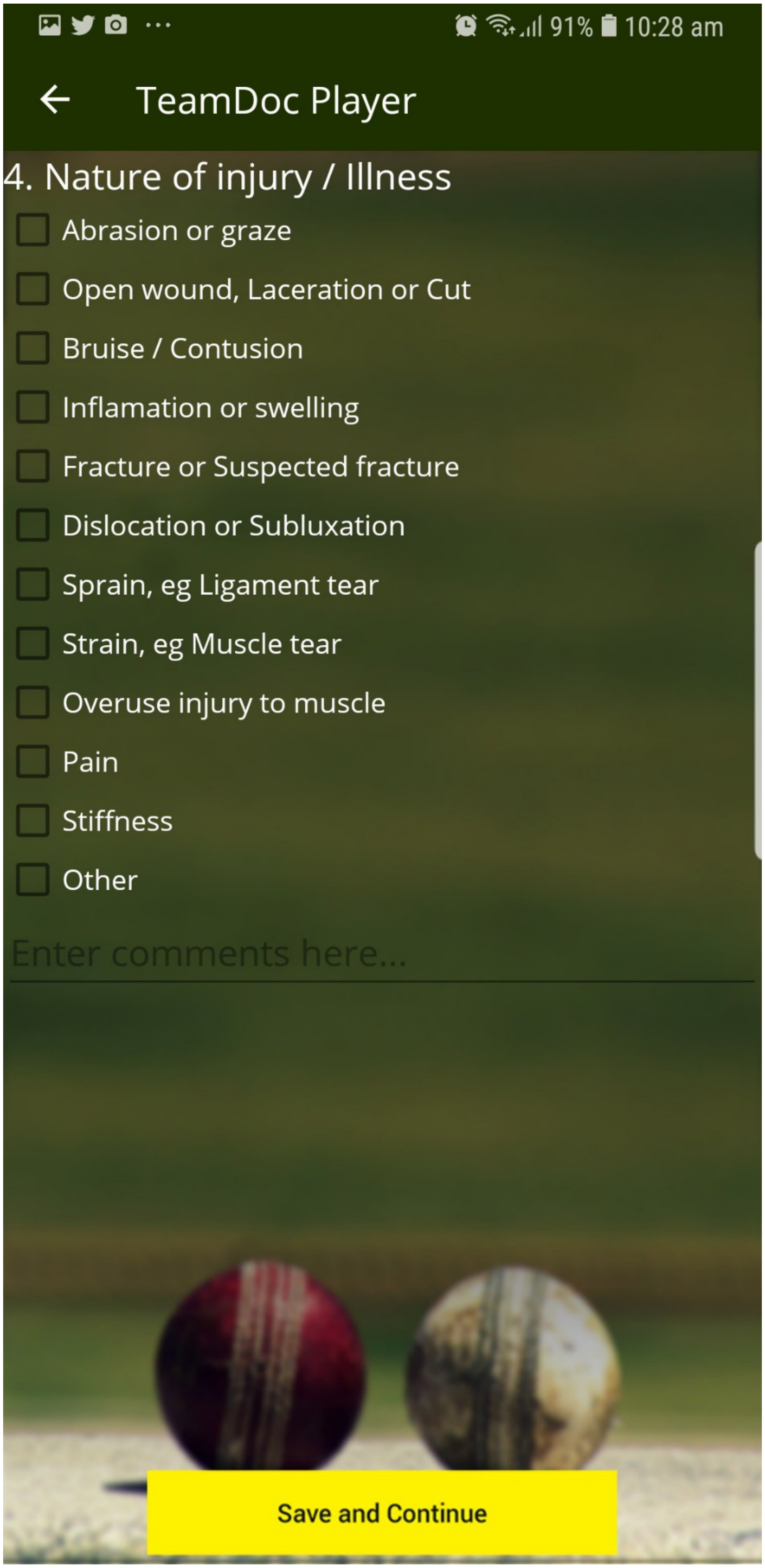
91% 10:28 am
TeamDoc Player
4. Nature of injury / Illness
Abrasion or graze
Open wound, Laceration or Cut
Bruise / Contusion
Inflamation or swelling
Fracture or Suspected fracture
Dislocation or Subluxation
Sprain, eg Ligament tear
Strain, eg Muscle tear
Overuse injury to muscle
Pain
Stiffness
Other
Enter comments here...
Save and Continue

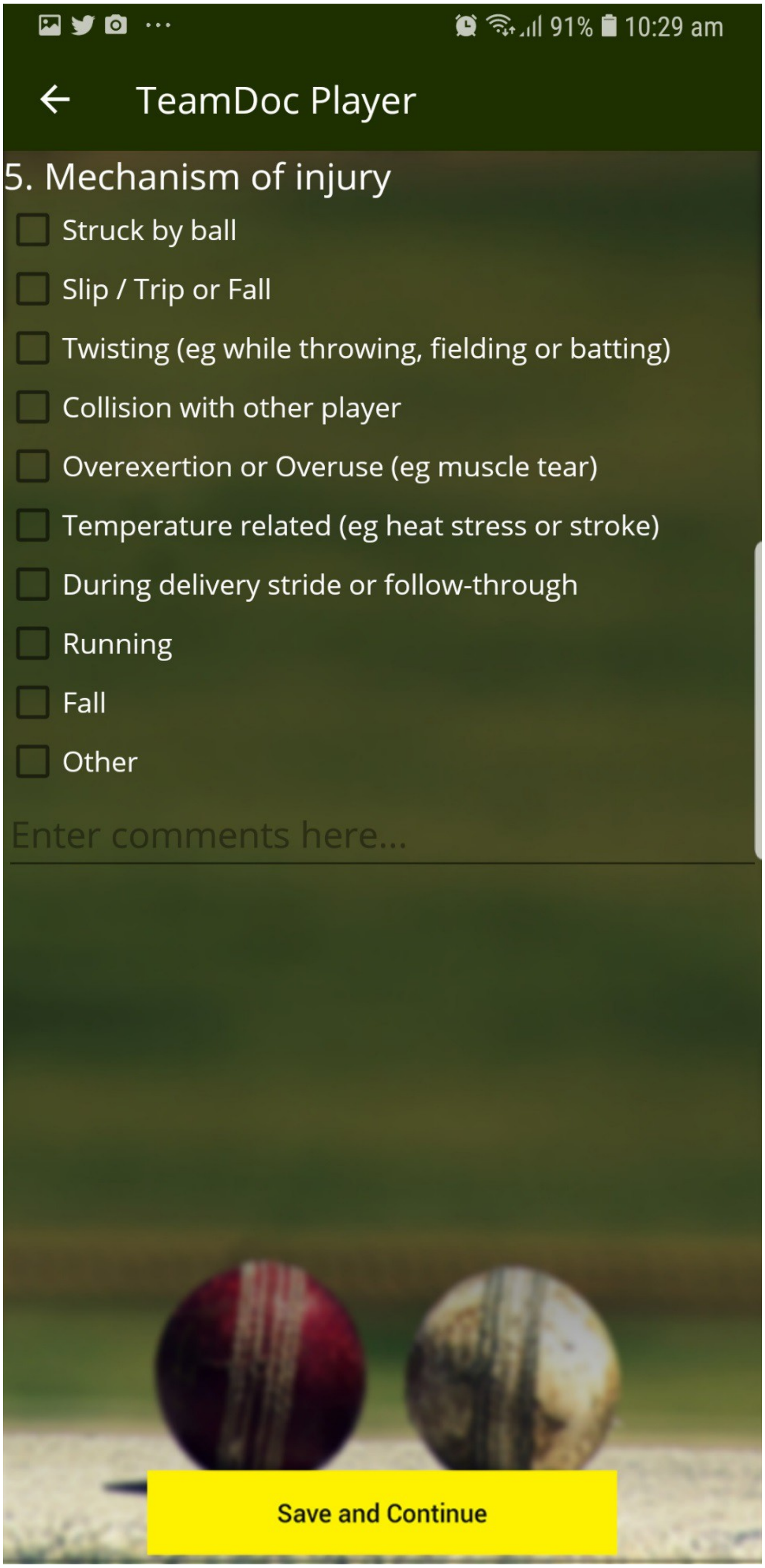
91% 10:29 am
TeamDoc Player
5. Mechanism of injury
Struck by ball
Slip / Trip or Fall
Twisting (eg while throwing, fielding or batting)
Collision with other player
Overexertion or Overuse (eg muscle tear)
Temperature related (eg heat stress or stroke)
During delivery stride or follow-through
Running
Fall
Other
Enter comments here...
Save and Continue

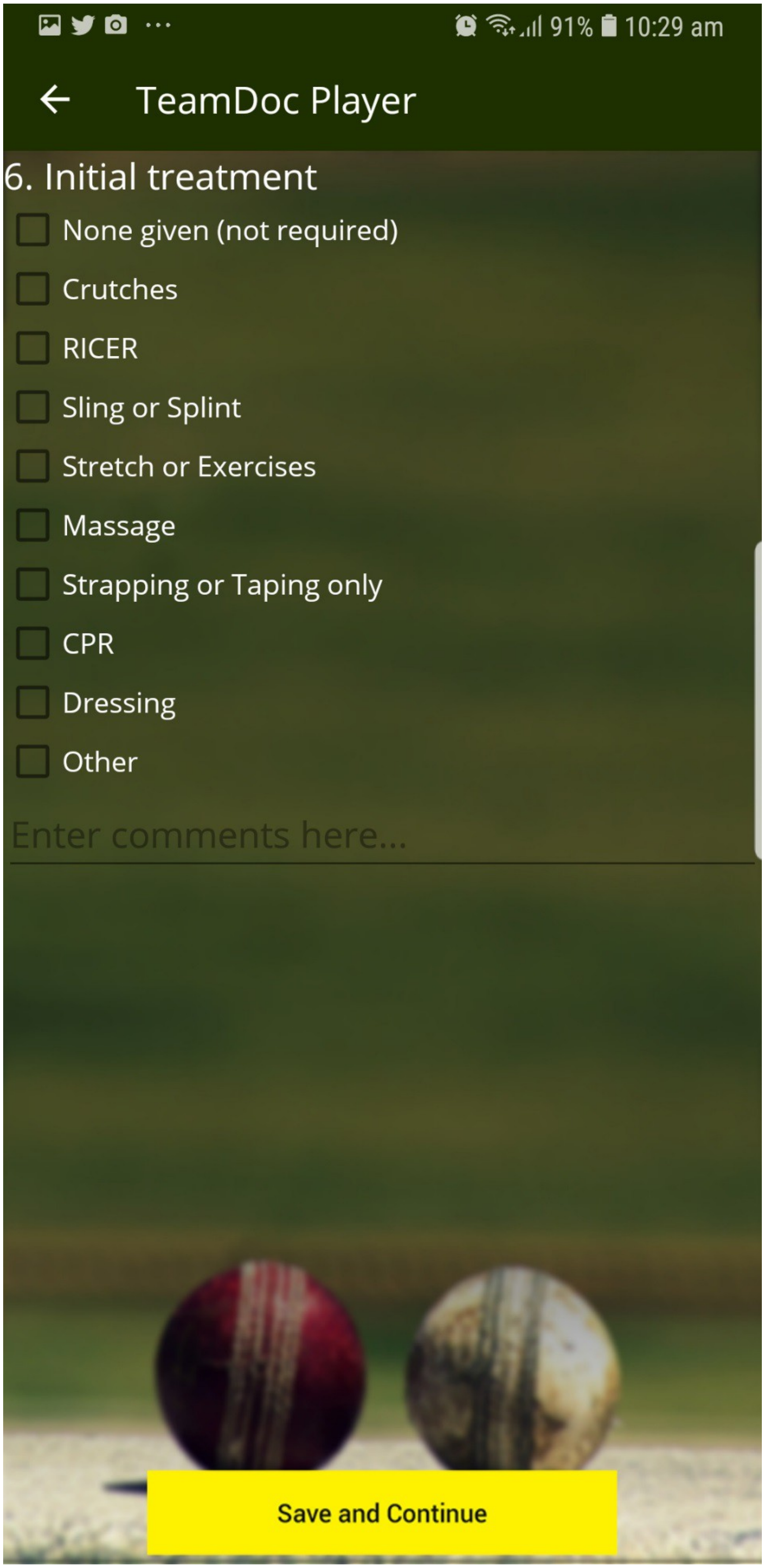
91% 10:29 am
TeamDoc Player
6. Initial treatment
None given (not required)
Crutches
RICER
Sling or Splint
Stretch or Exercises
Massage
Strapping or Taping only
CPR
Dressing
Other
Enter comments here...
Save and Continue

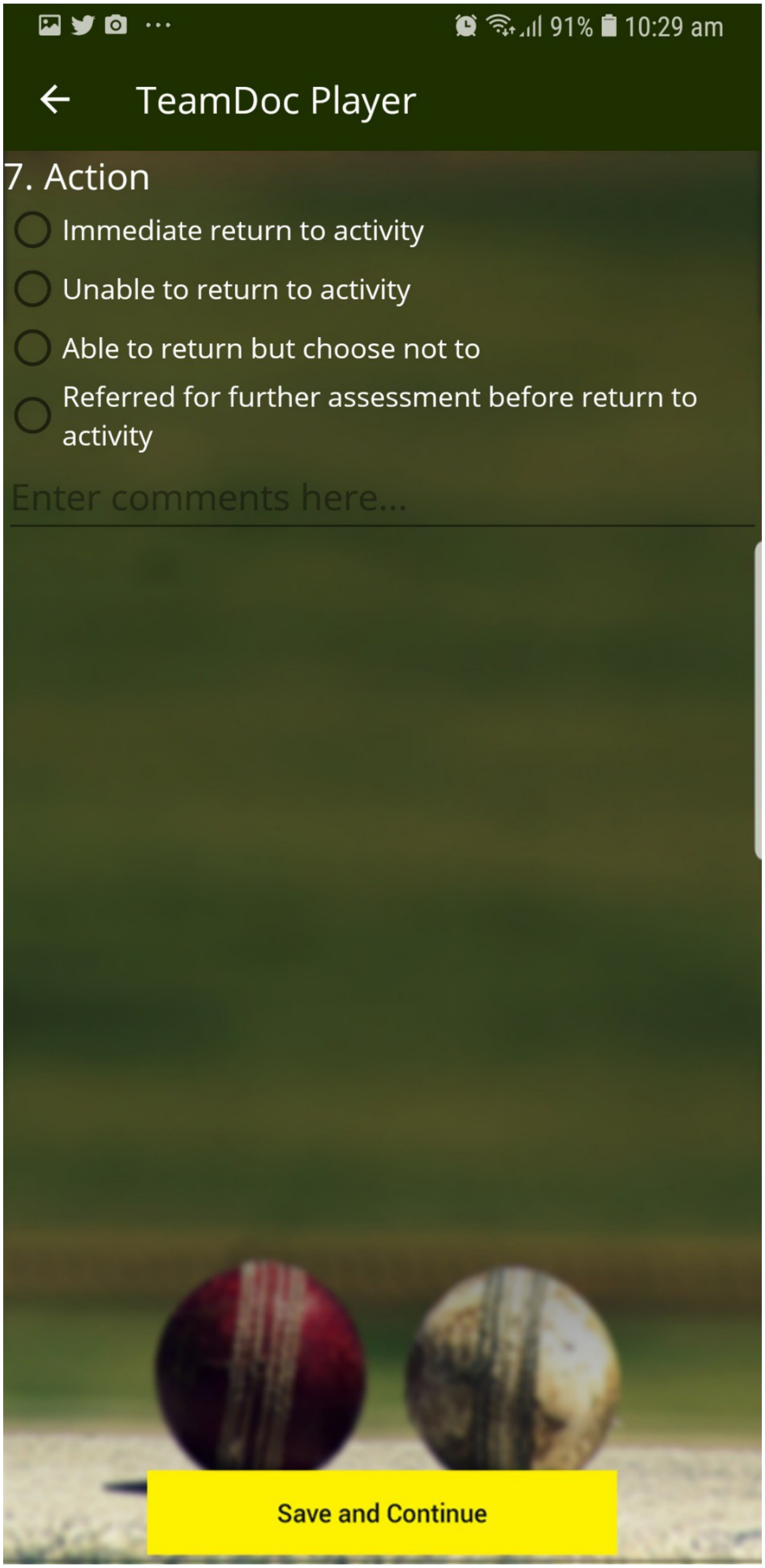
91% 10:29 am
TeamDoc Player
7. Action
Immediate return to activity
Unable to return to activity
Able to return but choose not to
Referred for further assessment before return to activity
Enter comments here...
Save and Continue

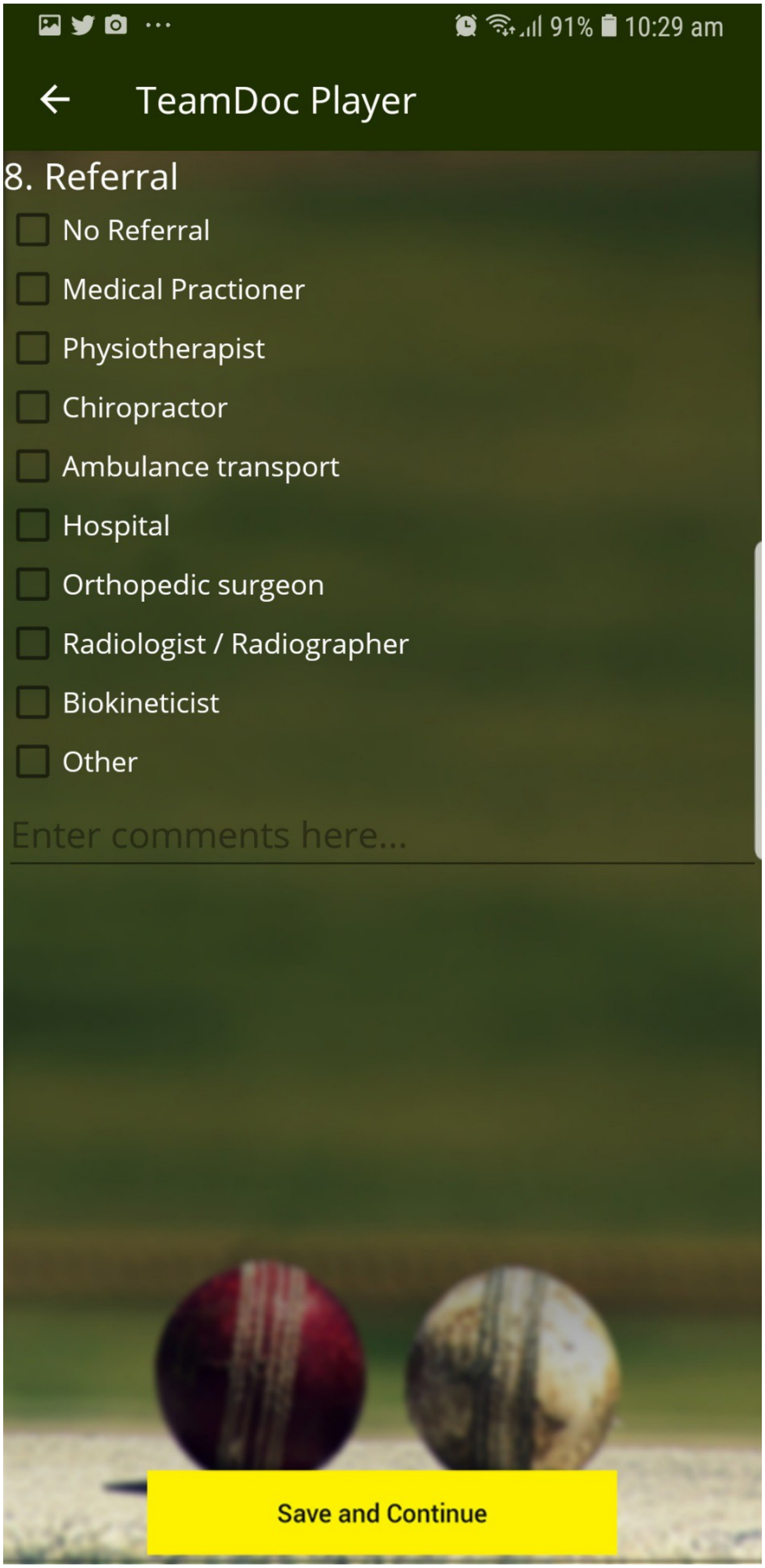
91% 10:29 am
TeamDoc Player
8. Referral
No Referral
Medical Practioner
Physiotherapist
Chiropractor
Ambulance transport
Hospital
Orthopedic surgeon
Radiologist / Radiographer
Biokineticist
Other
Enter comments here...
Save and Continue

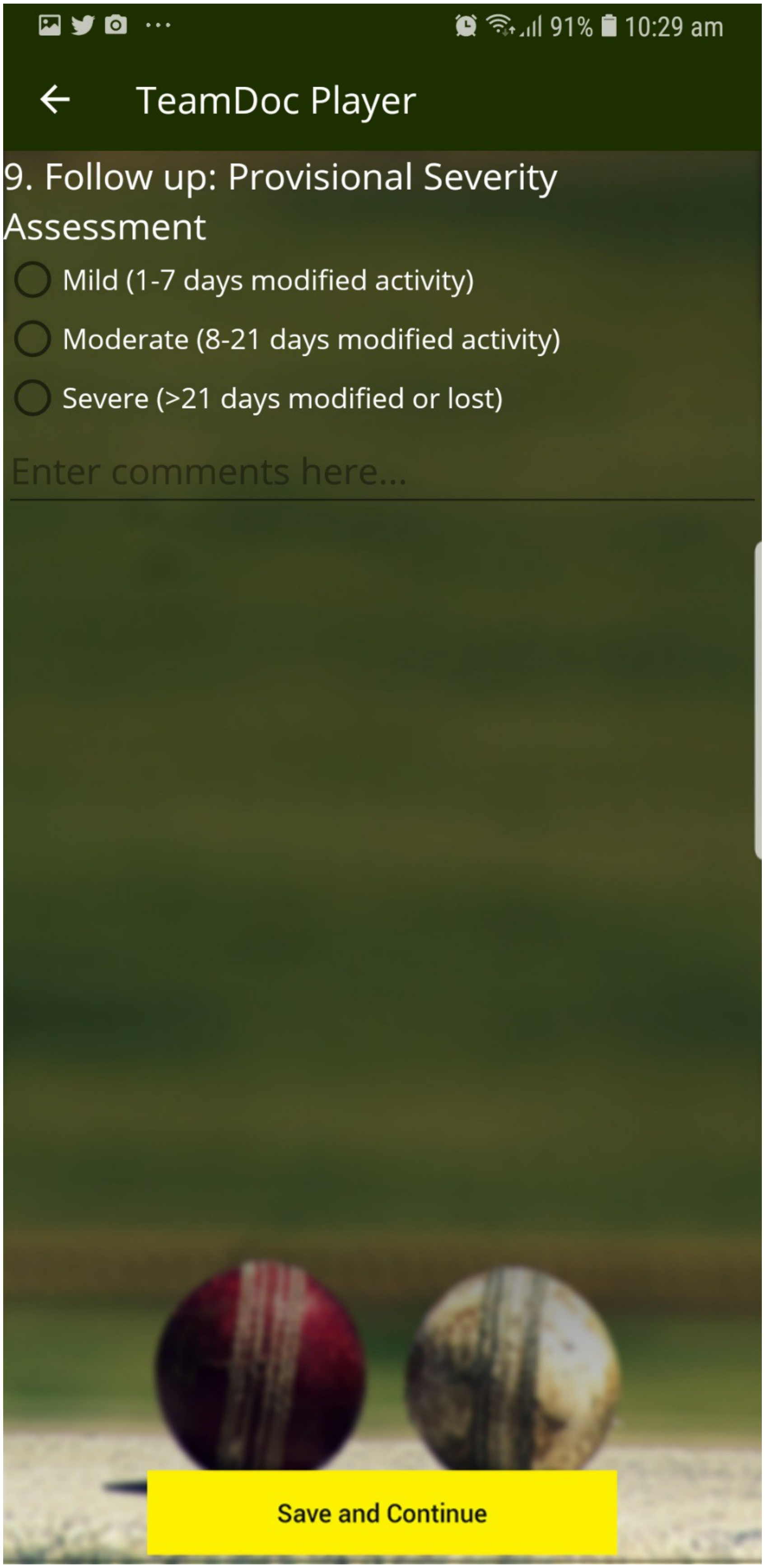
91% 10:29 am
TeamDoc Player
9. Follow up: Provisional Severity Assessment
Mild (1-7 days modified activity)
Moderate (8-21 days modified activity)
Severe (>21 days modified or lost)
Enter comments here...
Save and Continue

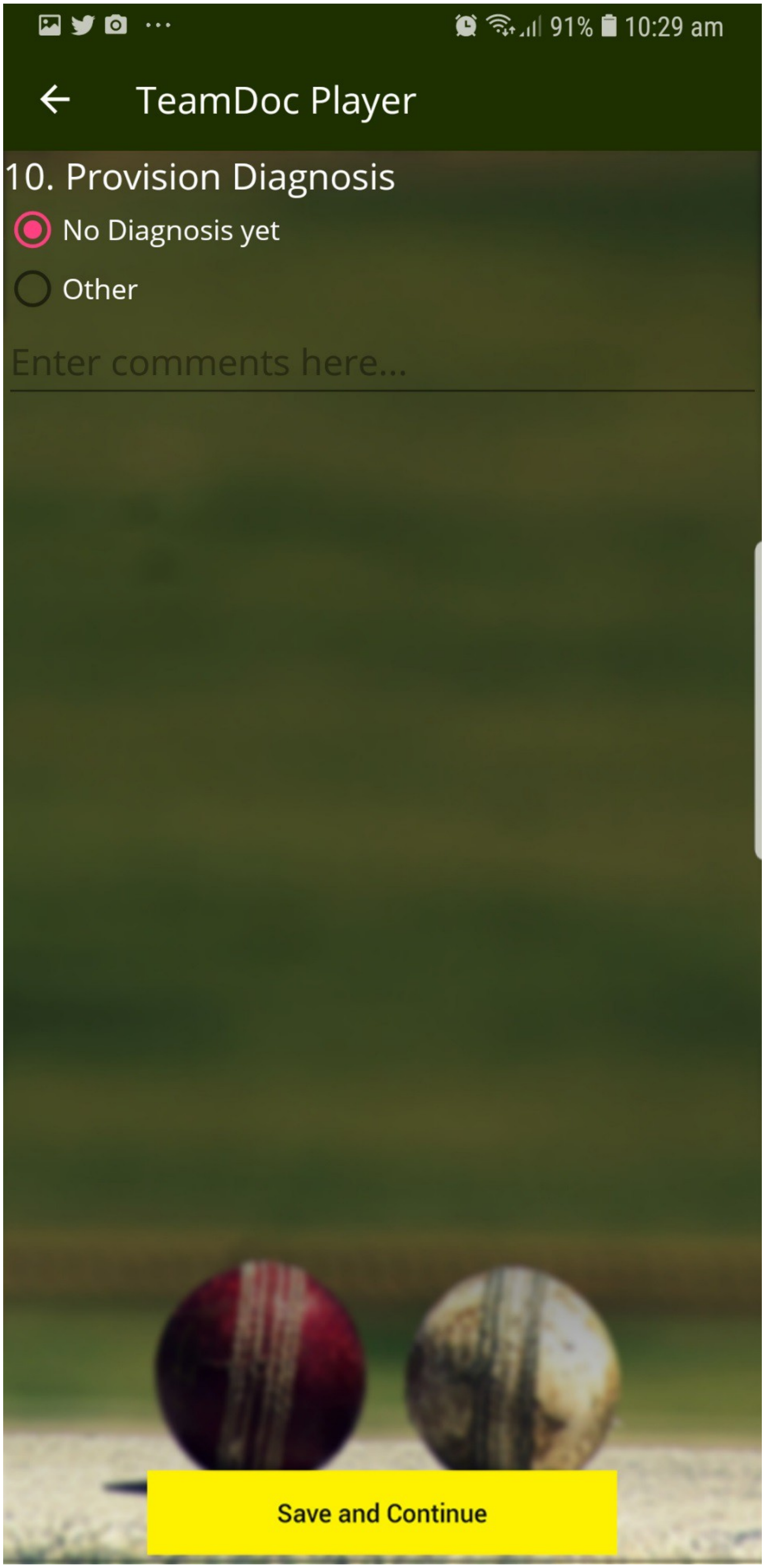
91% 10:29 am
TeamDoc Player
10. Provision Diagnosis
No Diagnosis yet
Other
Enter comments here...
Save and Continue

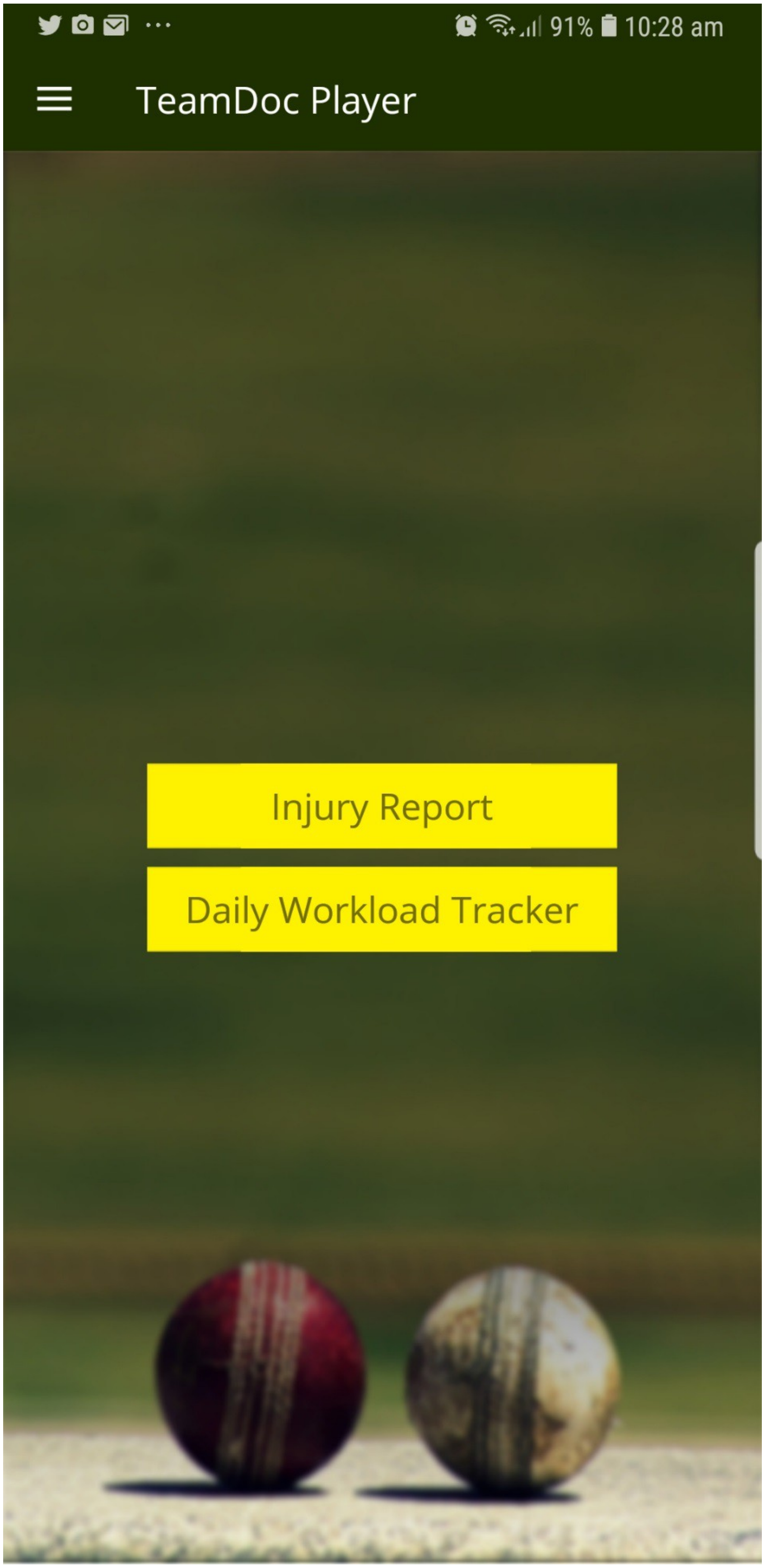
91% 10:28 am
TeamDoc Player
Injury Report
Daily Workload Tracker

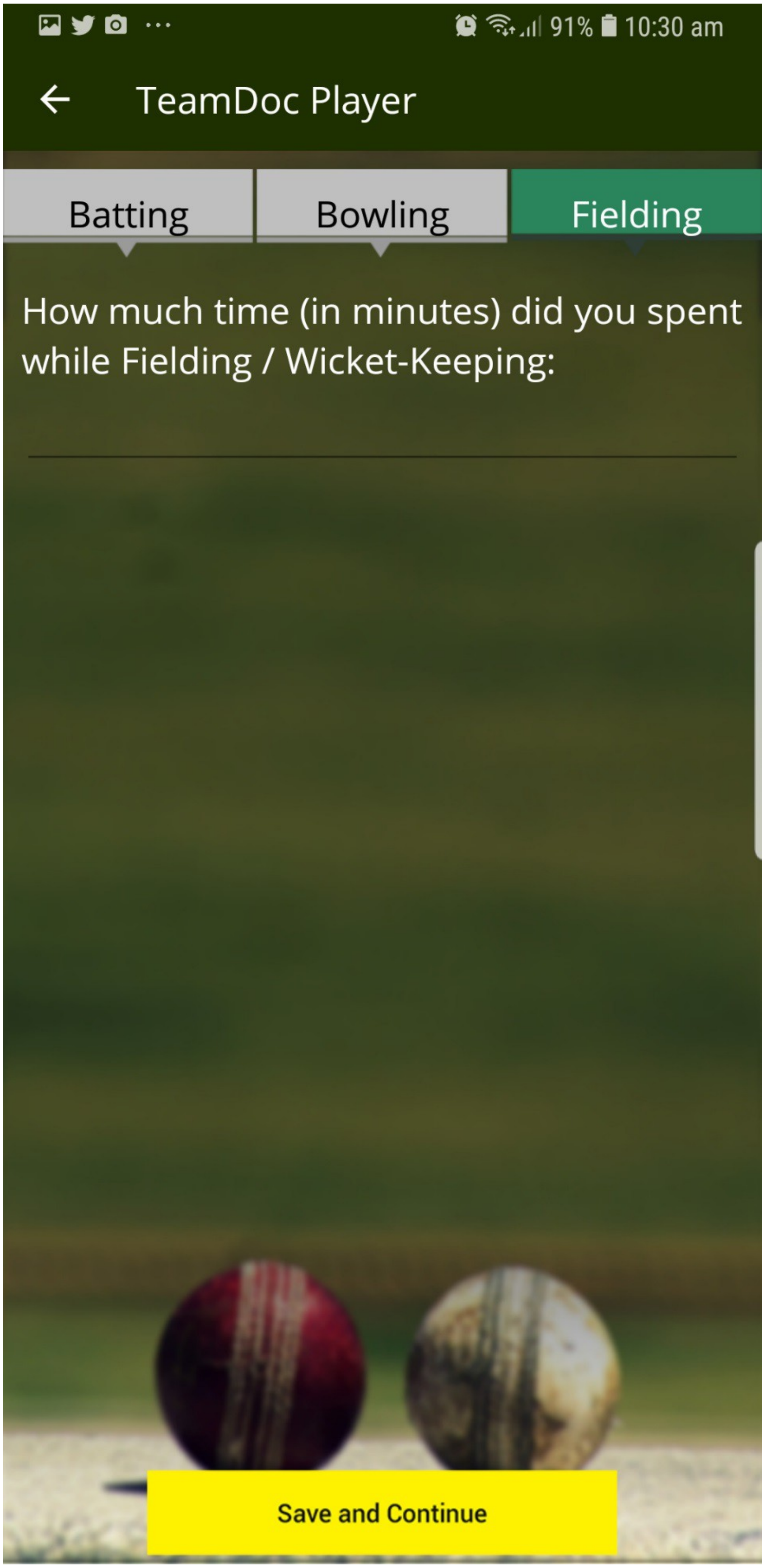
91% 10:30 am
TeamDoc Player
Batting
Bowling
Fielding
How much time (in minutes) did you spent while Fielding / Wicket-Keeping:
Save and Continue

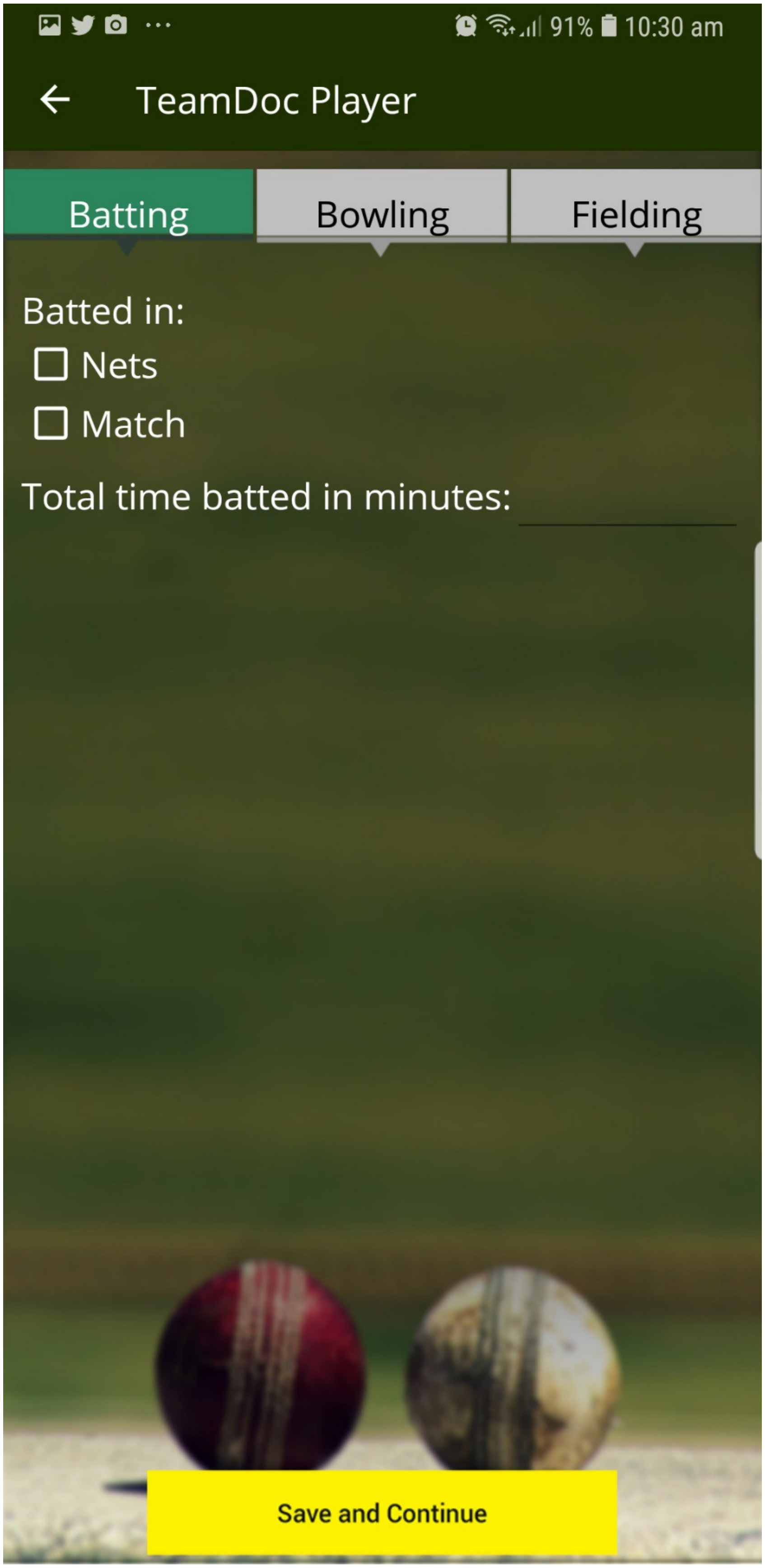
91% 10:30 am
TeamDoc Player
Batting
Bowling
Fielding
Batted in:
Nets
Match
Total time batted in minutes:
Save and Continue

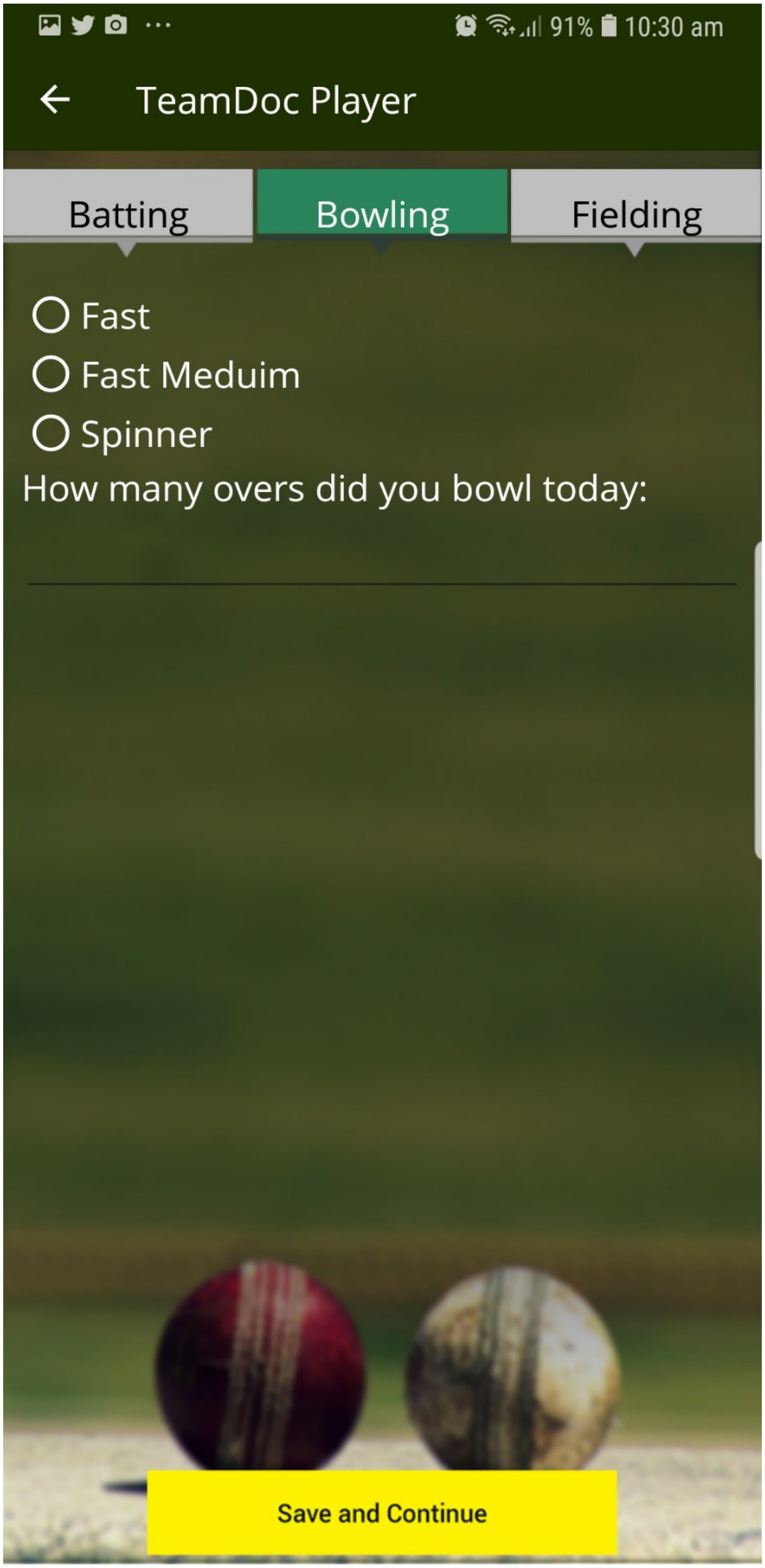
91% 10:30 am
TeamDoc Player
Batting
Bowling
Fielding
Fast
Fast Meduim
Spinner
How many overs did you bowl today:
Save and Continue
