# Supplementary material for: Design, Development, and Evaluation of an Injury Surveillance App for Cricket: Protocol and Qualitative Study
Source: JMIR Mhealth Uhealth. 2019 Jan 22;7(1):e10978. doi: 10.2196/10978 (PMC6362388; doi:10.2196/10978)
Supplement: Multimedia Appendix 3 [file mhealth_v7i1e10978_app3.pdf]

Appendix 3:

Modified Mobile Application Rating Scale (MARS)

App Classification

Name: ____ **Age:** _____________________________

Email: ____ **Phone:** ____________________________

Cricket Club: ________________________________

Role in the team (Batsman,Spin Bowler etc): ________________________________

How many years have you played club cricket: __________________________

Mobile Phone Model: __________________________ **Version:** _______________________________________

Platform: ☐ Phone ☐ Pad ☐ Android

App Quality Ratings

The Rating scale assesses app quality on four dimensions. All items are rated on a 5-point scale from "1.Inadequate" to "5.Excellent". Circle the number that most accurately represents the quality of the app component you are rating. Please use the descriptors provided for each response category.

SECTION A

Engagement – fun, interesting, customisable, interactive (e.g. sends alerts, messages, reminders, feedback, enables sharing), well-targeted to audience

1. **Entertainment: Is the app fun/entertaining to use? Does it use any strategies to increase engagement through entertainment (e.g. through gamification)?**

   1 Dull, not fun or entertaining at all
   2 Mostly boring
   3 OK, fun enough to entertain user for a brief time (< 5 minutes)
   4 Moderately fun and entertaining, would entertain user for some time (5-10 minutes total)
   5 Highly entertaining and fun, would stimulate repeat use

2. **Interest: Is the app interesting to use? Does it use any strategies to increase engagement by presenting its content in an interesting way?**

   1 Not interesting at all
   2 Mostly uninteresting
   3 OK, neither interesting nor uninteresting; would engage user for a brief time (< 5 minutes)
   4 Moderately interesting; would engage user for some time (5-10 minutes total)
   5 Very interesting, would engage user in repeat use

3. **Customisation: Does it provide/retain all necessary settings/preferences for apps features (e.g. sound, content, notifications, etc.)?**

   1 Does not allow any customisation or requires setting to be input every time
   2 Allows insufficient customisation limiting functions
   3 Allows basic customisation to function adequately
   4 Allows numerous options for customisation
   5 Allows complete tailoring to the individual's characteristics/preferences, retains all settings

4. **Interactivity: Does it allow user input, provide feedback, contain prompts (reminders, sharing options, notifications, etc.)? Note: these functions need to be customisable and not overwhelming in order to be perfect.**

   1 No interactive features and/or no response to user interaction
   2 Insufficient interactivity, or feedback, or user input options, limiting functions
   3 Basic interactive features to function adequately
   4 Offers a variety of interactive features/feedback/user input options
   5 Very high level of responsiveness through interactive features/feedback/user input options

5. **Target group: Is the app content (visual information, language, design) appropriate for your target audience?**

   1 Completely inappropriate/unclear/confusing
   2 Mostly inappropriate/unclear/confusing
   3 Acceptable but not targeted. May be inappropriate/unclear/confusing
   4 Well-targeted, with negligible issues
   5 Perfectly targeted, no issues found

A. Engagement mean score = ____________

SECTION B

Functionality – app functioning, easy to learn, navigation, flow logic, and gestural design of app

6. **Performance: How accurately/fast do the app features (functions) and components (buttons/menus) work?**

   1 App is broken; no/insufficient/inaccurate response (e.g. crashes/bugs/broken features, etc.)
   2 Some functions work, but lagging or contains major technical problems
   3 App works overall. Some technical problems need fixing/Slow at times
   4 Mostly functional with minor/negligible problems
   5 Perfect/timely response; no technical bugs found/contains a 'loading time left' indicator

7. **Ease of use: How easy is it to learn how to use the app; how clear are the menu labels/icons and instructions?**

   1 No/limited instructions; menu labels/icons are confusing; complicated
   2 Useable after a lot of time/effort
   3 Useable after some time/effort
   4 Easy to learn how to use the app (or has clear instructions)
   5 Able to use app immediately; intuitive; simple

8. **Navigation: Is moving between screens logical/accurate/appropriate/ uninterrupted; are all necessary screen links present?**

   1 Different sections within the app seem logically disconnected and random/confusing/navigation is difficult
   2 Usable after a lot of time/effort
   3 Usable after some time/effort
   4 Easy to use or missing a negligible link
   5 Perfectly logical, easy, clear and intuitive screen flow throughout, or offers shortcuts

9. **Gestural design: Are interactions (taps/swipes/pinches/scrolls) consistent and intuitive across all components/screens?**

   1 Completely inconsistent/confusing
   2 Often inconsistent/confusing
   3 OK with some inconsistencies/confusing elements
   4 Mostly consistent/intuitive with negligible problems
   5 Perfectly consistent and intuitive

B. Functionality mean score = ___________

SECTION C

Aesthetics – graphic design, overall visual appeal, colour scheme, and stylistic consistency

10. **Layout: Is arrangement and size of buttons/icons/menus/content on the screen appropriate or zoomable if needed?**

   1 Very bad design, cluttered, some options impossible to select/locate/see/read device display not optimised
   2 Bad design, random, unclear, some options difficult to select/locate/see/read
   3 Satisfactory, few problems with selecting/locating/seeing/reading items or with minor screen-size problems
   4 Mostly clear, able to select/locate/see/read items
   5 Professional, simple, clear, orderly, logically organised, device display optimised. Every design component has a purpose

11. **Graphics: How high is the quality/resolution of graphics used for buttons/icons/menus/content?**

1 Graphics appear amateur, very poor visual design - disproportionate, completely stylistically inconsistent
2 Low quality/low resolution graphics; low quality visual design – disproportionate, stylistically inconsistent
3 Moderate quality graphics and visual design (generally consistent in style)
4 High quality/resolution graphics and visual design – mostly proportionate, stylistically consistent
5 Very high quality/resolution graphics and visual design - proportionate, stylistically consistent throughout

12. **Visual appeal: How good does the app look?**

1 No visual appeal, unpleasant to look at, poorly designed, clashing/mismatched colours
2 Little visual appeal – poorly designed, bad use of colour, visually boring
3 Some visual appeal – average, neither pleasant, nor unpleasant
4 High level of visual appeal – seamless graphics – consistent and professionally designed
5 As above + very attractive, memorable, stands out; use of colour enhances app features/menus

C. Aesthetics mean score = ______________

App subjective quality assessment

SECTION D

13. Would you recommend this app to people who might benefit from it?

1	**Not at all**	I would not recommend this app to anyone
2		There are very few people I would recommend this app to
3	**Maybe**	There are several people whom I would recommend it to
4		There are many people I would recommend this app to
5	**Definitely**	I would recommend this app to everyone

14. How many times do you think you would use this app in the next 12 months if it was relevant to you?

1 **None**
2 1-2
3 3-10
4 10-50
5 >50

15. Would you pay for this app?

1 No
3 Maybe
5 Yes

16. What is your overall star rating of the app?

1	★	One of the worst apps I've used
2	★★	
3	★★★	Average
4	★★★★	
5	★★★★★	One of the best apps I've used

Scoring

App quality scores for

SECTION

A: Engagement Mean Score = ___________________________

B: Functionality Mean Score = ___________________________

C: Aesthetics Mean Score = ___________________________

App quality mean Score = ___________________________

App subjective quality Score = _________________________

App-specific

These items are used to assess the perceived impact of the app on the user's knowledge, attitudes, intentions to change as well as the likelihood of actual change in the target health behavior.

SECTION F

17. Awareness: This app is likely to increase awareness of the importance of addressing injury in cricket?

Strongly disagree | | | | Strongly Agree

1 2 3 4 5

18. Knowledge: This app is likely to increase knowledge/understanding of injury in cricket?

Strongly disagree | | | | Strongly Agree

1 2 3 4 5

19. Attitudes: This app is likely to change attitudes toward improving injury in cricket?

Strongly disagree | | | | Strongly Agree

1 2 3 4 5

20. Intention to change: This app is likely to increase intentions/motivation to address injury in cricket?

Strongly disagree | | | | Strongly Agree

1 2 3 4 5

21. Help seeking: Use of this app is likely to encourage further help seeking for my injury?

Strongly disagree | | | | Strongly Agree

1 2 3 4 5

22. Behaviour change: Use of this app is likely to help me understand my injury risk and seek help if injured?

Strongly disagree | | | | Strongly Agree

1 2 3 4 5

23. If you decide not to use this app what will be the possible reasons for it?

____

____

24. What improvements do you want to see in future versions of this app?

____

____
